# Association Between ^18^F-FDG PET/CT-Based SUV Index and Malignant Status of Persistent Ground-Glass Nodules

**DOI:** 10.3389/fonc.2021.594693

**Published:** 2021-03-24

**Authors:** Rong Niu, Yuetao Wang, Xiaoliang Shao, Zhenxing Jiang, Jianfeng Wang, Xiaonan Shao

**Affiliations:** ^1^ Department of Nuclear Medicine, The Third Affiliated Hospital of Soochow University, Changzhou, China; ^2^ Changzhou Key Laboratory of Molecular Imaging, The Third Affiliated Hospital of Soochow University, Changzhou, China; ^3^ Department of Radiology, The Third Affiliated Hospital of Soochow University, Changzhou, China

**Keywords:** adenocarcinoma of lung, risk factors, positron emission tomography computed tomography, fluorodeoxyglucose F18, ground-glass nodule

## Abstract

To explore the association between ^18^F-FDG PET/CT-based SUV index and malignant risk of persistent ground-glass nodules (GGNs). We retrospectively analyzed a total of 166 patients with GGN who underwent PET/CT examination from January 2012 to October 2019. There were 113 women and 53 men, with an average age of 60.8 ± 9.1 years old. A total of 192 GGNs were resected and confirmed by pathology, including 22 in benign group and 170 in adenocarcinoma group. They were divided into three groups according to SUV index tertiles: Tertile 1 (0.14–0.54), Tertile 2 (0.55–1.17), and Tertile 3 (1.19–6.78), with 64 GGNs in each group. The clinical and imaging data of all patients were collected and analyzed. After adjusting for the potential confounding factors, we found that the malignancy risk of GGN significantly decreased as the SUV index increased (OR, 0.245; 95%CI, 0.119–0.504; *P <*0.001), the average probability of malignant GGN was 89.1% (95% CI, 53.1–98.3%), 80.5% (95% CI, 36.7–96.7%), and 34.3% (95%CI, 9.5–72.2%) for Tertile 1 to Tertile 3. And the increasing trend of SUV index was significantly correlated with the reduction of malignant risk (OR, 0.099; 95%CI, 0.025–0.394; *P* = 0.001), especially between Tertile 3 versus Tertile 1 (OR, 0.064; 95%CI, 0.012–0.356; *P* = 0.002). Curve fitting showed that the SUV index was linearly and negatively correlated with the malignant risk of GGN. SUV index is an independent correlation factor for malignancy risk of GGN, the higher the SUV index, the lower the probability of GGN malignancy.

## Introduction

Ground-glass nodule (GGN) refers to the increased attenuation of the lung parenchyma without obscuring the inside bronchi and pulmonary vascular structures on CT images. Several studies have found that the biological behavior of GGN is significantly different from solid lung nodules. GGN has a higher risk of malignancy than solid nodules, especially persistent GGN (1, 2), but it can also result from granuloma, alveolar hemorrhage, or interstitial fibrosis (3). Malignant GGN often lacks typical imaging features, and its CT features often overlap with benign GGN, which makes the diagnosis very difficult. Moreover, the diagnostic methods, such as fiberoptic bronchoscopy and percutaneous lung biopsy under CT positioning, have high false-negative rates and certain side effects, so their application values are limited. ^18^F-FDG PET/CT is an accurate non-invasive examination method, which uses the metabolic differences between tumor tissues and normal tissues to diagnose and locate tumors.

For solid lung nodules, the maximum standard uptake value (SUV_max_) >2.5 was used as the diagnostic standard, with a sensitivity of 79–100% and a specificity of 60–100% (4). However, the diagnostic value of PET/CT for GGN is still controversial. Many studies report that GGN is different from solid nodules, and there are high false-negative rate and false-positive rate in PET imaging (5). Son et al. (6) found that the malignant GGN, which was early lung adenocarcinoma, usually showed low FDG uptake or no uptake, with an average SUV_max_ of 1.9 (range: 0.5–6.0). On the other hand, more and more studies have found that benign GGN often exhibits “pseudo” tumor features, showing high FDG uptake. McDermott et al. (7) also found that the FDG metabolism of malignant GGN was significantly lower than benign GGN. Therefore, we hypothesized that GGN was different from other solid tumors and might have unique glucose metabolism characteristics. The traditional criteria to distinguish between benign and malignant nodules do not apply to GGN. Therefore, the correlation between FDG metabolic parameters of GGN and its malignancy is particularly crucial for identifying malignant from benign GGN. However, there are currently very few reports on this topic.

In this study, we retrospectively analyzed the clinical data of patients with suspected lung adenocarcinoma manifesting as GGN who underwent ^18^F-FDG PET/CT examination before surgery and extracted their FDG uptake features. Since SUV_max_ is susceptible to changes in patients’ blood glucose, body weight, and imaging schemes, this study used the SUV index (GGN SUV_max_/liver SUV_mean_) as the PET metabolic parameter to analyze the correlation between SUV index and malignant status of GGN.

## Materials and Methods

### General Data

**Figure 1:**
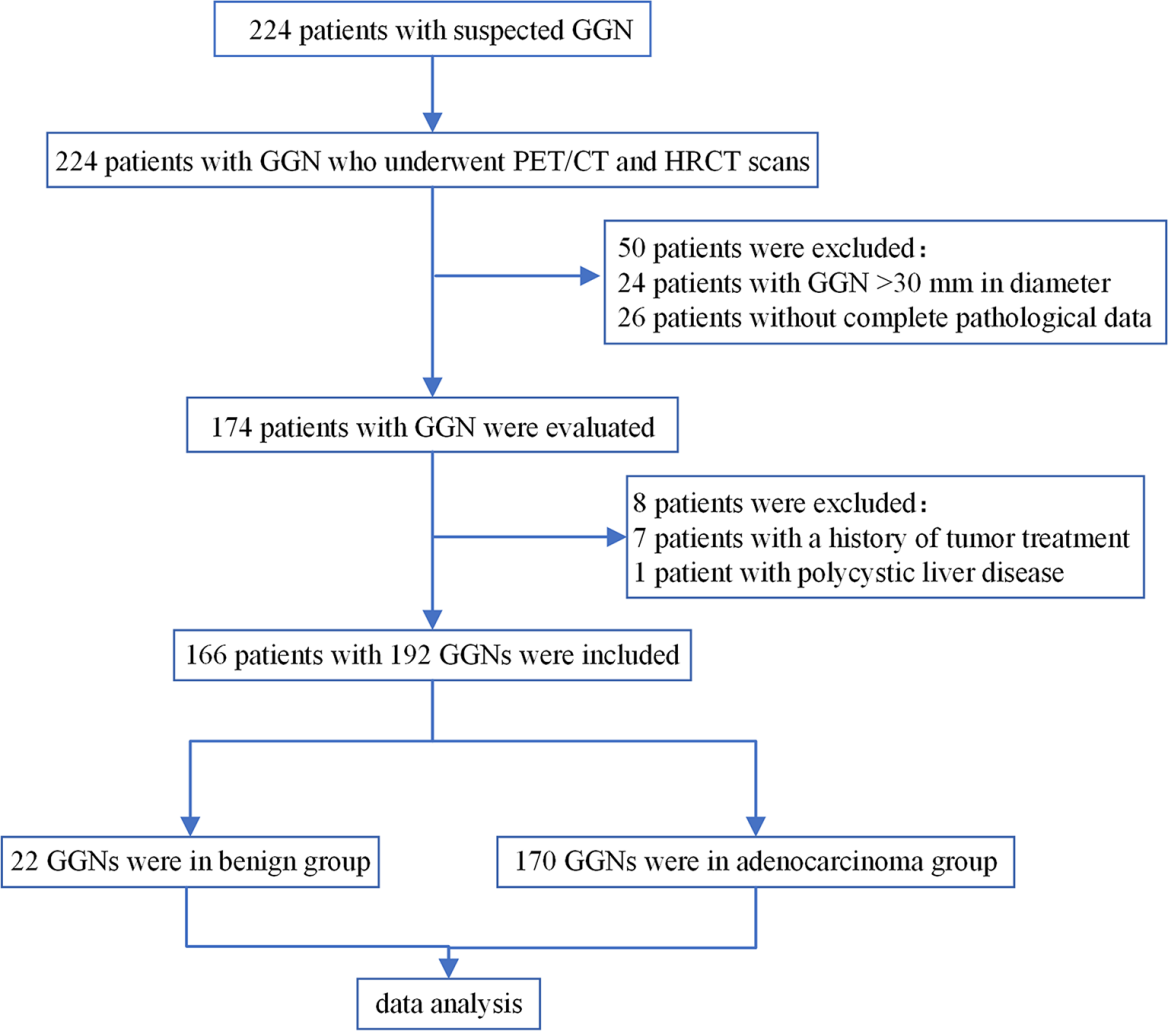
Flowchart of patient selection. GGN, ground-glass nodule.

This is a retrospective cohort study. We collected the clinical data of patients with persistent GGN who underwent ^18^F-FDG PET/CT examination in our hospital from January 2012 to October 2019. Persistent GGNs were defined as GGNs with no change or minor changes after CT follow-up for more than 3 months (8). Inclusion criteria: (1) Patients with Persistent GGNs; (2) Both PET/CT and HRCT scans were performed; (3) The diameter of GGN was ≤30 mm; (4) The surgery was completed within one month after PET/CT examination; (5) The pathological type of malignant GGN conformed to the new classification of lung adenocarcinoma published by IASLC/ATS/ERS in 2011 (9). Exclusion criteria: (1) Patients received anti-tumor therapy previously; (2) Fasting blood glucose was greater than 11.1 mmol/L; (3) Patients with severely impaired liver function. The patient selection process was shown in **Figure 1**.

A total of 166 patients were included in the study, of which 113 were female, and 53 were male, with an average age of (60.8 ± 9.1) years old. There were 110 cases with single GGN and 56 cases with multiple GGN. A total of 192 GGNs were surgically resected and confirmed by pathology: 22 were in benign group including two organizing pneumonia, one interstitial pneumonia, one pulmonary alveoli epithelium bronchial metaplasia, four fungal infections, three granulomas, and 11 other benign lesions; 170 were in adenocarcinoma group, including 143 invasive adenocarcinomas (IAC), 17 minimally invasive adenocarcinomas (MIA), five adenocarcinomas *in situ* (AIS), and five atypical adenomatous hyperplasias (AAH).

### Instruments and Imaging Method

Imaging equipment: German Siemens Biograph mCT (64) PET/CT scanner. Image acquisition: the patients were required to fast for 4–6 h before examination, and the height, weight, and blood sugar on the day of examination were recorded; PET/CT whole-body imaging was performed 1 h after the imaging agent (^18^F-FDG, 3.70–5.55 MBq/kg) injection, and was imaged first with low-dose CT and then PET scan. PET imaging was performed at 2 min/bed, about six to seven beds per patient according to their heights. The TrueX + TOF (ultraHD-PET) method was used for image reconstruction, with two iterations and 21 subsets; the matrix was 200 × 200, and the image acquisition mode was 3D. The post-processing workstation TrueD system (Siemens) was used for image evaluation (lung window width 1,200 HU, window level −600 HU, mediastinal window width 350 HU, window level 40 HU).

Then, the HRCT scan was performed on the GGN site under a breath-hold condition. The collection and reconstruction conditions were as follows: the tube voltage was 140 kV, and the tube current was automatically adjusted by caredose software according to human anatomy and tissue density; the rotation time was 0.5 s, the pitch was 0.6, the layer thickness was 1.0 mm, the layer interval was 0.5 mm, the matrix was 512 × 512, and the lung and mediastinal windows were set as before.

### Image Analysis

All images were examined and recorded by two nuclear medicine physicians with more than five years of work experience without knowing the pathology. When their opinions were not unified, they would discuss together to reach the consensus. PET image indicators include nodule SUV_max_, liver SUV_mean_, and SUV index. The measurement of nodule SUV_max_ required the fusion image of CT lung window and PET: a circular region of interest (ROI) was selected, which completely covered the nodule. For liver SUV_mean_ measurement, a circular ROI with a diameter of about 60 mm was selected on the right liver lobe. SUV index was the ratio of nodule SUV_max_ to liver SUV_mean_.

**Figure 2:**
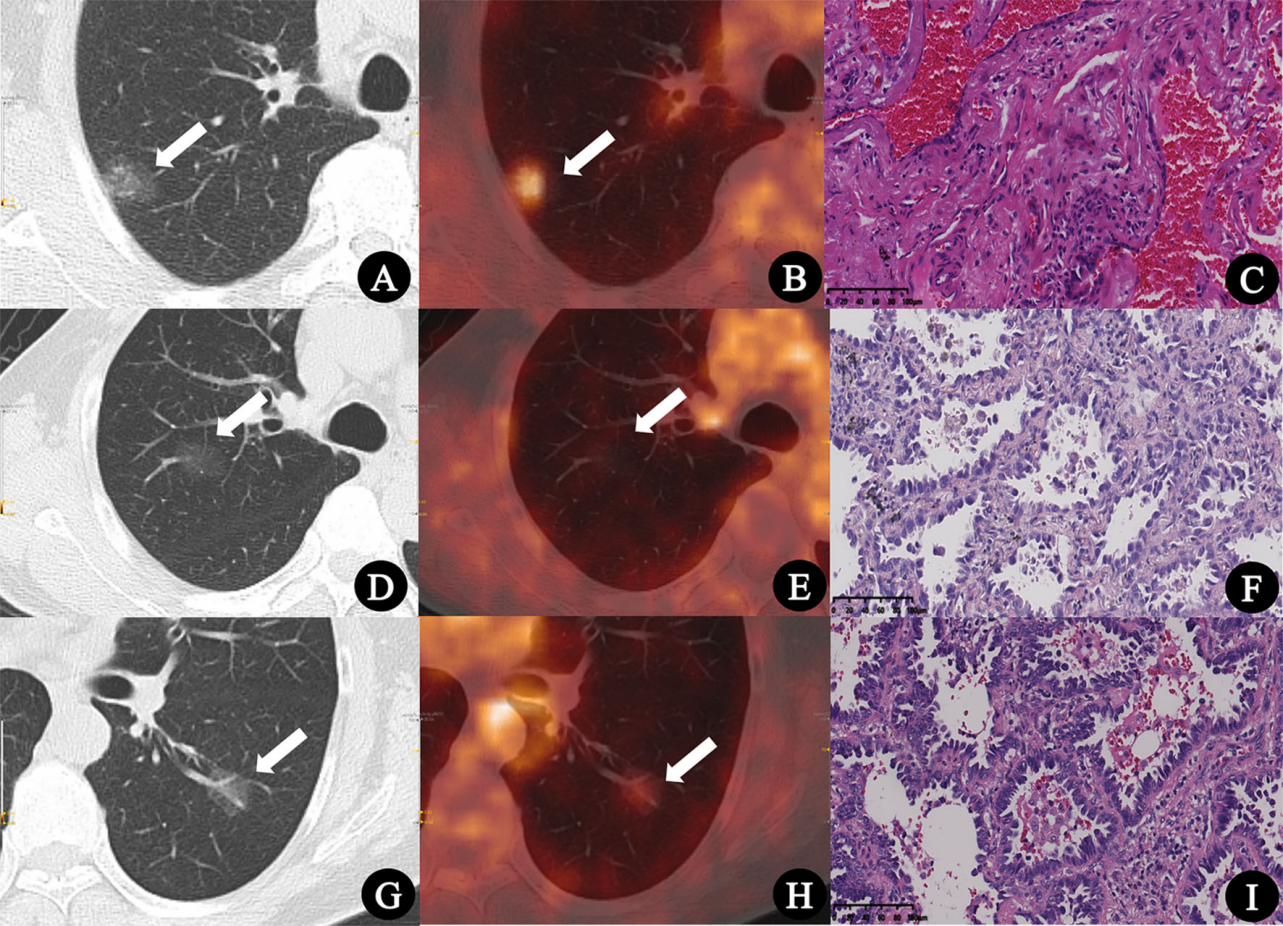
**(A–C)** Inflammatory pseudotumor in a 77-year-old man. **(A)** CT lung window image showed a pure ground-glass nodule with a diameter of 22.1 mm in the right upper lobe (arrow). **(B)** PET/CT fusion image showed that the SUV_max_ of the lesion was 3.2, and the SUV index was 1.28 (arrow). **(C)** Pathologic findings indicated fibrous tissue hyperplasia, hyperemia, and inflammatory cell infiltration (HE × 200). **(D–F)** Minimally invasive adenocarcinoma in a 74-year-old man. **(D)** CT lung window image showed a pure ground-glass nodule with a diameter of 20.9 mm in the right upper lobe (arrow). **(E)** PET/CT fusion image showed that the SUV_max_ of the lesion was 0.6, and the SUV index was 0.22 (arrow). **(F)** Pathologic findings indicated well-differentiated minimally invasive adenocarcinoma (HE × 200). **(G–I)** Invasive adenocarcinoma in a 55-year-old woman. **(G)** CT lung window image showed a pure ground-glass nodule with a diameter of 18.8 mm in the left lower lung lobe (arrow). **(H)** PET/CT fusion image showed that the SUV_max_ of the lesion was 1.4, and the SUV index was 0.52 (arrow). **(I)** Pathologic findings indicated moderately differentiated invasive adenocarcinoma (HE × 200).

HRCT indicators include: nodule number (single, multiple), nodule location (periphery, center), nodule type [pure ground-glass nodules (pGGN), mixed ground-glass nodules (mGGN)], margin (smooth, lobulated), shape (round/quasi-round, irregular), abnormal air bronchogram (dilated, distorted, or cutoff), vacuolar sign, pleural indentation, vascular convergence, nodule diameter (D_GGN_, the largest cross-sectional diameter of the nodules), diameter of nodule solid component (D_solid_, the largest cross-sectional diameter of the solid components), proportion of solid components (CTR, the ratio of D_solid_ to D_GGN_), the average CT value of ground glass component (CT_GGO_, the average CT value of the GGN ground glass component: the circular ROI was applied to measure the CT value of GGO in the lung window of three different layers, and then calculated their average value; the solid components, blood vessels, dilated bronchia, and vacuoles were avoided during measurement), the average CT value of normal lung parenchyma (CT_LP_, the average CT value of normal lung tissues around GGN: a circular ROI was used to measure the average of three measurements on different lung winder layers), ΔCT_GGO-LP_ (difference between CT_GGO_ and CT_LP_). All the average value of the measurements from two physicians was recorded. Three typical cases are shown in **Figure 2**.

### Statistical Analysis

The baseline features of all nodules and the summary statistics stratified by SUV index tertiles were expressed as the frequency and proportion of categorical variables, mean ± SD, or median and quartile. Chi-square test (categorical variable), one-way ANOVA (normally distributed continuous variable), and Kruskal–Wallis test (skewed continuous variable) were used to analyze the differences between groups. The intraclass correlation coefficient (ICC) was used to analyze the data consistency between the two observers.

Univariate logistic regression analysis was used to calculate the relationship between different nodule features and the malignancy risk of GGN. The generalized linear models with a logit link were used to test the independent and comprehensive effects of SUV index on GGN malignant status (binary variables). We calculated unadjusted and adjusted estimates using exact methods and asymptotic methods, respectively. Covariates were included as potential confounders in the final models if they changed the estimates of SUV index on malignant risk of GGN by more than 10% or were significantly associated with malignant risk of GGN (*P <*0.1). We calculated the odds ratio (OR) and 95% confidence interval (CI). Tertile 1 of SUV index was used as the reference.

Stratified analysis was used to assess further the association between SUV index and malignant risk of GGN in each subgroup. Generalized additive models (GAM) were used to test the nonlinear relationship between the malignant risk of GGN and SUV index. It helped to find the nonlinear relationship, and determine whether there was a threshold effect and whether it was appropriate to use general linear regression. All data were analyzed using R software (version 3.4.3, http://www.R-project.org). *P <*0.05 was considered statistically significant.

### Ethics

This study was approved by the institutional ethics committee for retrospective analysis [(2018) KD 013] and did not require informed consent.

## Results

### General Information Among the SUV Index Tertiles

192 GGNs were divided into three groups according to SUV index tertiles: Tertile 1 (0.14–0.54), Tertile 2 (0.55–1.17), and Tertile 3 (1.19–6.78), with 64 GGNs in each group. The clinical characteristics and PET/CT parameters across different groups were compared and listed in **Table 1**. There was no statistical difference in age, gender, GGN number and location between different groups (all *P >*0.05); while smoking history, nodules types, shapes, margins, abnormal air bronchogram, vacuolar sign, pleural indentation, vascular convergence, D_GGN_, D_solid_, CTR, CT_GGO_, and ΔCT_GGO-LP_ showed significant differences between groups (*P <*0.05 for all). The GGN malignancy probability of the entire cohort in this study was 88.5%. From SUV index Tertile 1 to Tertile 3, the malignant proportions of GGN were 89.1, 95.3, and 81.2%, respectively, which were not significantly different (*P* = 0.05). Two observers had great consistency in the results of PET/CT and HRCT parameter measurement (ICC: 0.907–0.999, all *P <*0.001).

**Table 1:** Comparison of clinical characteristics and PET/CT parameters of GGN across different SUV index tertiles.

Characteristics	Total (n = 192)	Tertile 1 (n = 64)	Tertile 2 (n = 64)	Tertile 3 (n = 64)	*P*-value
Age (years)	60.2 ± 8.9	58.3 ± 9.0	62.0 ± 7.9	60.4 ± 9.6	0.064
Gender					0.411
Female	131 (68.2%)	47 (73.4%)	44 (68.8%)	40 (62.5%)	
Male	61 (31.8%)	17 (26.6%)	20 (31.2%)	24 (37.5%)	
Smoking history	36 (18.8%)	10 (15.6%)	6 (9.4%)	20 (31.2%)	0.005
GGN number					0.758
Single	110 (57.3%)	35 (54.7%)	39 (60.9%)	36 (56.2%)	
Multiple	82 (42.7%)	29 (45.3%)	25 (39.1%)	28 (43.8%)	
Nodule type					<0.001
pGGN	66 (34.4%)	39 (60.9%)	20 (31.2%)	7 (10.9%)	
mGGN	126 (65.6%)	25 (39.1%)	44 (68.8%)	57 (89.1%)	
Location					1.000
Periphery	186 (96.9%)	62 (96.9%)	62 (96.9%)	62 (96.9%)	
Center	6 (3.1%)	2 (3.1%)	2 (3.1%)	2 (3.1%)	
Shape					0.001
Round/quasi-round	115 (59.9%)	50 (78.1%)	32 (50.0%)	33 (51.6%)	
Irregular	77 (40.1%)	14 (21.9%)	32 (50.0%)	31 (48.4%)	
Margin					<0.001
Smooth	106 (55.2%)	50 (78.1%)	34 (53.1%)	22 (34.4%)	
Lobulated	86 (44.8%)	14 (21.9%)	30 (46.9%)	42 (65.6%)	
Abnormal air bronchogram	139 (72.4%)	34 (53.1%)	51 (79.7%)	54 (84.4%)	<0.001
Vacuolar sign	29 (15.1%)	5 (7.8%)	8 (12.5%)	16 (25.0%)	0.019
Pleura indentation	111 (57.8%)	19 (29.7%)	50 (78.1%)	42 (65.6%)	<0.001
Vascular convergence	179 (93.2%)	54 (84.4%)	63 (98.4%)	62 (96.9%)	0.002
D_GGN_(mm)	18.9 ± 6.7	13.5 ± 5.0	21.4 ± 5.8	21.9 ± 5.6	<0.001
D_solid_(mm)	7.0 ± 6.5	2.6 ± 4.3	7.2 ± 6.5	11.1 ± 5.6	<0.001
CTR	0.3 ± 0.3	0.2 ± 0.2	0.3 ± 0.3	0.5 ± 0.2	<0.001
CT_GGO_(HU)	−462.8 ± 132.3	−534.3 ± 127.4	−440.0 ± 133.1	−414.1 ± 104.9	<0.001
ΔCT_GGO-LP_(HU)	398.1 ± 122.6	336.5 ± 115.7	415.4 ± 129.1	442.5 ± 96.9	<0.001
Pathology					0.050
Benign	22 (11.5%)	7 (10.9%)	3 (4.7%)	12 (18.8%)	
Adenocarcinoma	170 (88.5%)	57 (89.1%)	61 (95.3%)	52 (81.2%)	

The results are expressed as mean ± (SD)/N (%).

pGGN, pure ground-glass nodule; mGGN, mixed ground-glass nodule; D_GGN_, diameter of the GGN; D_solid_, diameter of the solid component; CTR, D_solid_/D_GGN_; CT_GGO_, attenuation value of the GGO component on CT; ΔCT_GGO-LP_, CT_GGO_ − CT_LP_.

### Crude Association Between Nodular Features and Malignant Risk of GGN

We used malignant GGN (early lung adenocarcinoma) as the dependent variable (Y = 1) and 21 nodular features, including SUV index as the independent variables to perform the univariate logistic regression analysis. The results showed that age, gender, smoking history, pleural indentation, D_GGN_, and SUV index were all possible related factors of early lung adenocarcinoma (*P <*0.10 for all). The specific data are shown in **Table 2**.

**Table 2:** Crude association between nodular features and malignant risk of GGN.

Characteristics	Statistics	OR (95%CI)	*P*-value
Age (years)	60.2 ± 8.9	1.055 (1.004–1.109)	0.034
Age group (years)			0.035
<60	81 (42.2%)	1.0	
≥60	111 (57.8%)	2.690 (1.070–6.761)	
Gender			0.005
Female	131 (68.2%)	1.0	
Male	61 (31.8%)	0.272 (0.109–0.679)	
Smoking history	36 (18.8%)	0.345 (0.132–0.900)	0.030
GGN number			0.524
Single	110 (57.3%)	1.0	
Multiple	82 (42.7%)	1.349 (0.538–3.385)	
Nodule type			0.789
pGGN	66 (34.4%)	1.0	
mGGN	126 (65.6%)	0.878 (0.339–2.273)	
Location			0.988
Periphery	186 (96.9%)	1.0	
Center	6 (3.1%)	2099572.789 (0.000-Inf)	
Shape			0.402
Round/quasi-round	115 (59.9%)	1.0	
Irregular	77 (40.1%)	1.500 (0.581–3.870)	
Margin			0.199
Smooth	106 (55.2%)	1.0	
Lobulated	86 (44.8%)	1.860 (0.722–4.793)	
Abnormal air bronchogram	139 (72.4%)	1.983 (0.793–4.958)	0.143
Vacuolar sign	29 (15.1%)	0.776 (0.242–2.484)	0.669
Pleura indentation	111 (57.8%)	11.032 (3.139–38.780)	<0.001
Vascular convergence	179 (93.2%)	1.445 (0.299–6.995)	0.647
D_GGN_	18.9 ± 6.7	1.068 (0.997–1.144)	0.063
D_solid_	7.0 ± 6.5	1.028 (0.958–1.104)	0.437
CTR	0.3 ± 0.3	0.592 (0.134–2.619)	0.490
CTR (0.5)			0.606
CTR ≤0.5	123 (64.1%)	1.0	
CTR >0.5	69 (35.9%)	0.788 (0.318–1.950)	
CTR (0.25)			0.402
CTR ≤0.25	77 (40.1%)	1.0	
CTR >0.25	115 (59.9%)	0.667 (0.258–1.720)	
CT_GGO_	−462.8 ± 132.3	1.001 (0.997–1.004)	0.733
ΔCT_GGO-LP_	398.1 ± 122.6	1.000 (0.997–1.004)	0.888
SUV index	1.1 ± 1.0	0.547 (0.377–0.795)	0.002

The results are expressed as mean ± (SD)/N (%).

Inf: the model failed because of the small sample size.

pGGN, pure ground-glass nodule; mGGN, mixed ground-glass nodule; D_GGN_, diameter of the GGN; D_solid_, diameter of the solid component; CTR, D_solid_/D_GGN_; CT_GGO_, attenuation value of the GGO component on CT; ΔCT_GGO-LP_, CT_GGO_ - CT_LP_; SUV index, GGN SUV_max_/liver SUV_mean_.

### Multivariate Regression for the Association Between SUV Index and Malignant Risk of GGN

**Table 3** shows the results of univariate and multivariate logistic regression analysis on continuous SUV index and SUV index tertiles. Non-adjusted covariates were equivalent to single factor logistic regression analysis. The preliminarily adjusted (Adjust I) covariates included smoking history, pleural indentation, and D_GGN_, and fully adjusted (Adjust II) covariates included age, gender, smoking history, margin, abnormal air bronchogram, pleural indentation, and D_GGN_. In the regression equations of Non-adjusted, Adjust I and Adjust II covariates for continuous variables, the increase of SUV index significantly reduced the malignant risk of GGN, with ORs of 0.547, 0.378 and 0.245, respectively (all *P <*0.01); however, for the SUV index tertile-group, the increasing trend of SUV index was not significantly correlated with the reduction of GGN malignant risk (*P* = 0.101). In the regression equations of only Adjust I and Adjust II covariates, the increasing trend of SUV index was significantly correlated with the reduction of GGN malignant risk (OR was 0.225 and 0.099, *P* = 0.007 and 0.001, respectively), especially comparing Tertile 3 versus Tertile 1 (OR was 0.171 and 0.064, *P* = 0.011 and 0.002).

**Table 3:** Multivariate regression for the association between SUV index and malignant risk of GGN.

SUV index	Non-adjusted		Adjust I		Adjust II	
	OR (95%CI)	*P*-value	OR (95%CI)	*P-*value	OR (95%CI)	*P*-value
Total	0.547 (0.377–0.795)	0.002	0.378 (0.215–0.665)	0.001	0.245 (0.119–0.504)	<0.001
Tertile 1 (0.14–0.54)	1.0		1.0		1.0	
Tertile 2 (0.55–1.17)	2.497 (0.616–10.124)	0.200	0.669 (0.139–3.226)	0.617	0.506 (0.086–2.959)	0.449
Tertile 3 (1.19–6.78)	0.532 (0.195–1.454)	0.219	0.171 (0.044–0.663)	0.011	0.064 (0.012–0.356)	0.002
*P* for trend	0.478 (0.198–1.155)	0.101	0.225 (0.076–0.669)	0.007	0.099 (0.025–0.394)	0.001

Non-adjusted model adjust for: none.

Adjust I model adjust for: smoking history, pleural indentation, and D_GGN_.

Adjust II model adjust for: age, gender, smoking history, margin, abnormal air bronchogram, pleural indentation, and D_GGN_.

### Curve Fitting

**Figure 3:**
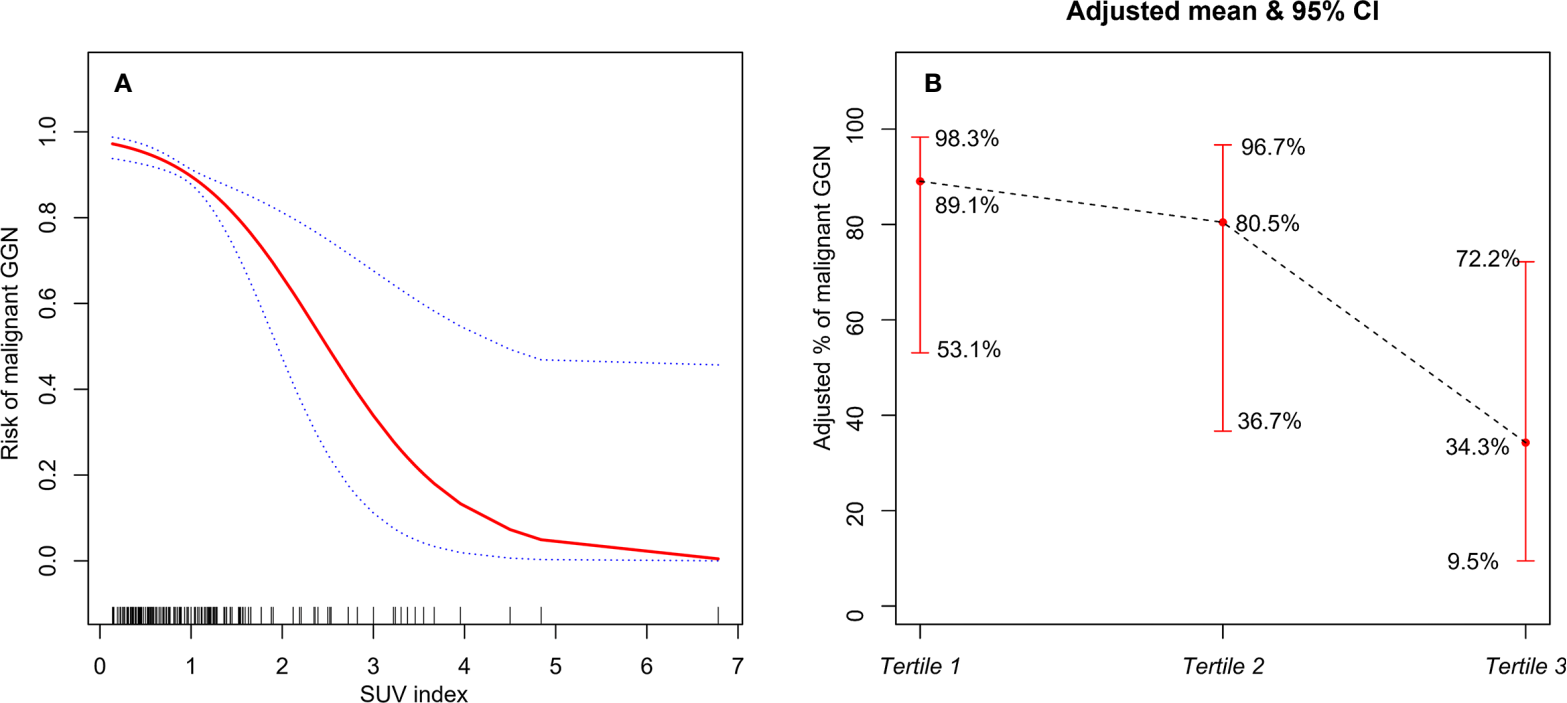
**(A)** The relationship between SUV index and malignant risk of GGN (the solid red line indicates the fitted line of the probability of malignant GGN and SUV index; the blue dotted line is the 95% confidence interval). Adjusted for: age, gender, smoking history, margin, abnormal air bronchogram, pleural indentation, and D_GGN_. **(B)** The relationship between SUV index tertiles and malignant risk of GGN (the black dotted line indicates the fitted line of the probability of malignant GGN and SUV index tertiles; the red line is the 95% confidence interval). Adjusted for: age, gender, smoking history, margin, abnormal air bronchogram, pleural indentation, and D_GGN_.

GAM was used to test the relationship between SUV index and malignant risk of GGN. The results showed that, after adjusting age, gender, smoking history, margin, abnormal air bronchogram, pleural indentation, and D_GGN_, the probability of malignant GGN gradually decreased with the increase of SUV index, and they showed approximate linear relationship (degree of freedom: 1.268, *P <*0.001, **Figure 3A**). If SUV index tertiles were used, the average probability of malignant GGN decreased gradually with the increase of SUV index level, which was 89.1% (95% CI, 53.1–98.3%), 80.5% (95% CI, 36.7–96.7%), and 34.3% (95%CI, 9.5–72.2%) for Tertile 1 to Tertile 3, respectively (**Figure 3B**). Tertile 3 had the lowest average probability of malignant GGN.

### Stratified Analysis

**Figure 4:**
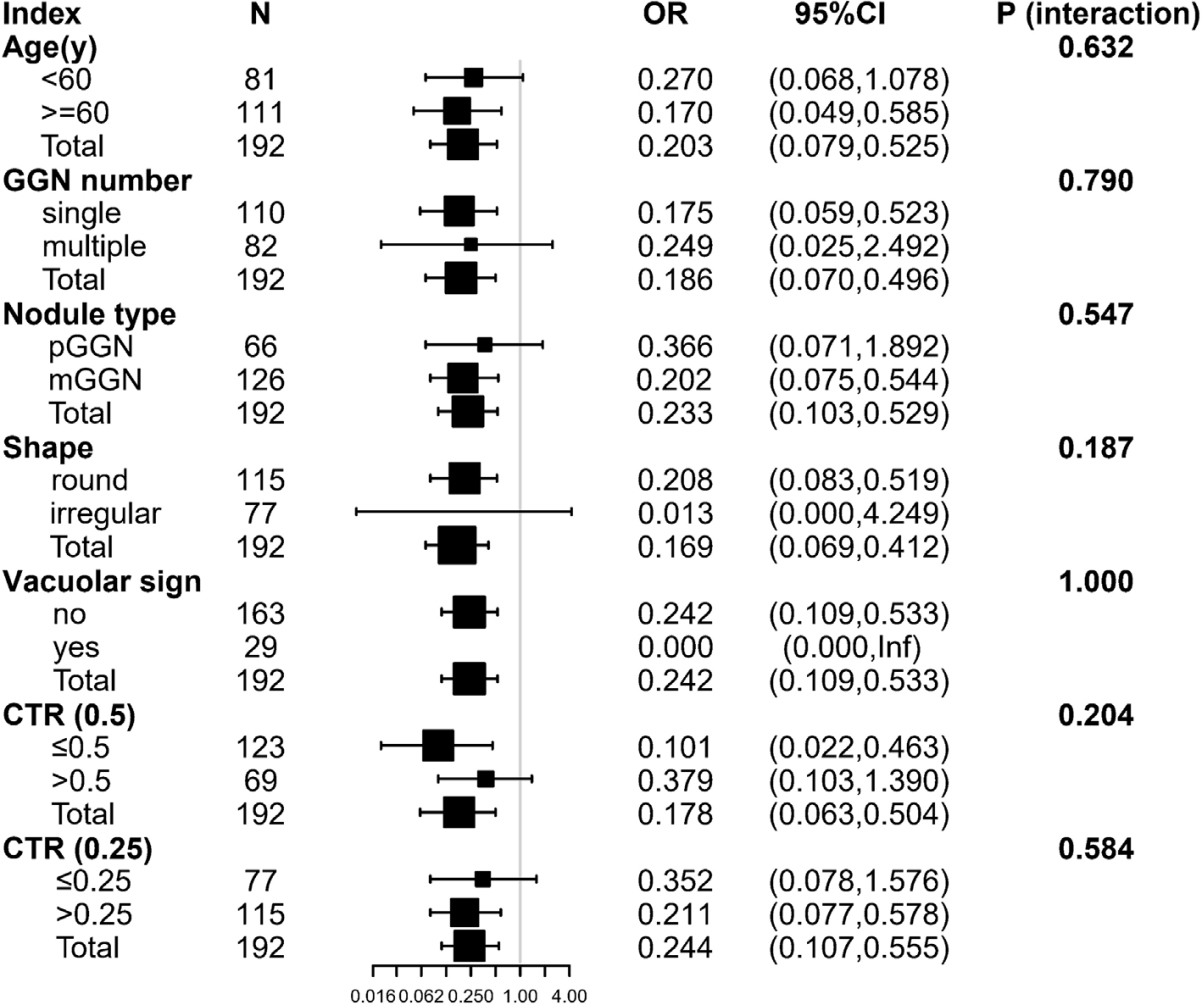
The stratification analysis of the association between SUV index and malignant risk of GGN. Inf: the model failed because of the small sample size. Adjusted for: age, gender, smoking history, margin, abnormal air bronchogram, pleural indentation, and D_GGN._ The following variables were excluded because they had <20 obs: nodule location, vascular convergence. pGGN, pure ground-glass nodule; mGGN, mixed ground-glass nodule; CTR, D_solid_/D_GGN_; D_GGN_, diameter of the GGN; D_solid_, diameter of the solid component.

Stratified analysis was used to assess further the association between SUV index and malignant risk of GGN in each subgroup. There was no variable (including age group, GGN number, nodule type, shape, vacuolar sign, CTR group) significantly changed the association between SUV index and GGN malignant status (*P* for interaction: 0.187–1.000) (**Figure 4**).

## Discussion

^18^F-FDG PET/CT scan has high application value in tumor diagnosis, staging, prognosis, and efficacy evaluation (10). It has become the primary imaging approach for evaluating lung cancer (3, 11). In general, the higher the FDG uptake, the more likely the lesion is malignant and aggressive. However, in recent years, many studies found that PET/CT had limited diagnostic value for GGN, with the high false-negative rate (57–90%) (5, 12), indicating that the characteristics of GGN glucose metabolism are different from solid lung cancer. In order to explore the relationship between SUV and malignant status of GGN, we retrospectively analyzed the data of patients with GGN who underwent preoperative PET/CT examination of suspicious lung adenocarcinoma. The results showed that SUV index is an independent correlation factor for malignant risk of GGN; the higher the SUV index, the lower the probability of GGN malignancy.

This study first explored the possible related factors that affected SUV index. The results showed that D_GGN_, D_solid_, CTR, CT_GGO_, ΔCT_GGO-LP_, and nodule types were significantly different in groups with different levels of SUV index. Consistently, several studies have also confirmed that the nodule size is an essential factor affecting SUV, and they are positively correlated: the larger the nodule diameter, the higher the SUV (6). D_solid_ and CTR represent the size and proportion of solid components in GGN. It has been reported that compared to the lung nodules with CTR <50%, the lung nodules with more solid components have significantly higher SUV_max,_ and the SUV_max_ of mGGN is higher than pGGN (6). The CT value reflects the number and density of cells in the lesion. The higher the CT value, the greater the density, and the number of abnormal cells in a unit space, so the FDG uptake will also be increased. In general, the SUV of solid nodules is higher than GGN (5). Also, we found that the morphological characteristics of nodules, such as pleural indentation, shape, vascular convergence, and smoking history were different in different SUV index groups, and these characteristics were also critical indicators to distinguish benign and malignant GGN.

Univariate analysis showed that age, gender, smoking history, pleural indentation, D_GGN,_ and SUV index might be related to early lung adenocarcinoma. Patients over 60 years old and non-smoker females had a significantly increased risk of GGN malignancy, which is consistent with the previous reports (13, 14). D_GGN_ is a risk factor for predicting early lung adenocarcinoma: the larger the nodule diameter, the higher the probability of GGN malignancy; this result is consistent with Lee et al. (15, 16). The Fleischner Society Guidelines (13) also point out that the nodule size is one of the essential parameters to distinguish between benign and malignant GGN. For GGNs that are difficult to identify, the nodule property should be further clarified by observing the nodule diameter changes during follow-up. SUV index is an influencing factor of malignant risk for GGN, but the difference in malignancy probability between different SUV index tertiles was not significant (*P* = 0.05). Thus, we reasoned that the smoking history, pleural indentation, and D_GGN_, which affected both SUV index and malignant status of GGN, were the potential confounding factors. This might be the reason why there was no significant statistical difference in the probability of GGN malignancy among the three tertile groups.

In order to control the influence of confounding factors, we established multiple regression equations. After fully correcting for the confounding factors, we finally confirmed that the SUV index was an independent factor for malignant status of GGN: the risk of malignant GGN significantly reduced with the increase of SUV index. Curve fitting showed an approximately linear negative correlation between SUV index and malignant status of GGN, and this correlation was not affected by age, GGN number, GGN type, shape, vacuolar sign, and CTR level.

It is well known that ^18^F-FDG, as a glucose analog, has an affinity for glucose receptors on the cell surface. Due to the increased expression of glucose transporter-1 in diseased tissues, the FDG uptake of cells is also increased (17). This process reflects the characteristic glucose metabolism of malignant tissues (18). Therefore, the malignant tumor is identified according to its significantly increased level of glucose metabolism compared to benign lesions and normal adjacent tissues. However, ^18^F-FDG is not a tumor-specific imaging agent. The chemotactic effect of inflammatory factors and the aggregation of inflammatory cells can also activate transporter proteins of inflammatory cells and promote FDG uptake by diseased tissues. Therefore, tuberculosis, interstitial pneumonia, cryptococcal infections, and some other benign diseases may also show increased FDG metabolism (19–21).

On the other hand, the early lung adenocarcinoma manifesting as GGN has features like low density, slow growth, and a high degree of differentiation; these inert characteristics often lead to low or no FDG uptake during PET/CT imaging (22, 23). Another study showed that glucose transporters are not overexpressed in early-stage lung adenocarcinoma, but the sodium glucose transporter 2, which does not transport ^18^F-FDG, is present in early-stage carcinoma (24). Chun et al. (25) reported that malignant GGNs demonstrated significantly lower FDG uptake at PET than benign GGNs. Our study comprehensively analyzed the influencing factors of SUV index and malignant status of GGN and verified the independent role of the two, which have not been reported before.

This study still has limitations (1): Since the enrolled cases were all patients with clinically suspected lung adenocarcinoma, not from screening data, the benign GGN sample size was relatively small. In future studies, the number of benign cases should be further expanded; (2) Although this study confirmed that there was a negative linear correlation between SUV index and malignant status of GGN, there were many confounding factors. Thus, for GGN diagnosis, it is still necessary to combine SUV index with a variety of clinical and imaging features for comprehensive evaluation; (3) It is worth noting that, although AAH was benign, this study classified it into adenocarcinoma group. The reason is that AAH is different from general inflammation and does not disappear after follow-up. It has been shown (26) that AAH to IAC is a sequential development process; thus, it is more reasonable to include AAH in the adenocarcinoma group.

In conclusion, this study showed that, in the ^18^F-FDG PET/CT imaging of persistent GGN, the SUV index was an independent correlation factor for malignancy risk of GGN, the higher the SUV index, the lower the probability of malignancy. When the SUV index ≥1.19, GGN was more likely to be benign lesions. Understanding and recognizing the relationship between SUV index and malignant status of GGN, which helps to improve the accuracy of diagnosis. PET/CT may be helpful in the diagnosis of persistent GGN or GGN patients seeking active treatment.

## Data Availability Statement

The original contributions presented in the study are included in the article/supplementary material. Further inquiries can be directed to the corresponding authors.

## Ethics Statement

This study was approved by the institutional ethics committee for retrospective analysis [(2018) KD 013] and did not require informed consent. Written informed consent for participation was not required for this study in accordance with the national legislation and the institutional requirements.

## Author Contributions

RN and XNS contributed to the study concepts and to the study design. RN and ZXJ contributed to data acquisition and reconstructions. XLS, RN, XNS, ZXJ, and JFW contributed to the data analyses and interpretation. XNS contributed to the statistical analysis. XNS, RN, and YTW contributed to the manuscript preparation and manuscript editing and reviewing. All authors read and approved the final manuscript. All authors contributed to the article and approved the submitted version.

## Funding

This study was supported by Young Talent Development Plan of Changzhou Health Commission (CZQM2020012); Key Laboratory of Changzhou High-tech Research Project (Grant No. CM20193010); and Changzhou Sci&Tech Program (Grant No. CJ20180022).

## Conflict of Interest

The authors declare that the research was conducted in the absence of any commercial or financial relationships that could be construed as a potential conflict of interest.
